# Impact of Advanced Age on Posttransplant Infections and Outcomes in Solid Organ Transplant Recipients: A Multicenter Cohort Study

**DOI:** 10.1111/tid.70232

**Published:** 2026-05-06

**Authors:** Sibel Altunisik Toplu, Vildan Avkan Oguz, Esra Tanyel, Imran Hasanoglu, Bengu Tatar, Tuba Turunc, Gule Cinar, Yesim Uygun Kizmaz, Gulnur Kul, Adem Kose, Muammer Celik, Adam Uslu, Ebru Oruc, Ezgi Erdal Karakas, Nilay Danis, Yelda Deligoz Bildaci, Sezai Yilmaz, Yasar Bayindir, Hande Arslan

**Affiliations:** ^1^ Department of Infectious Diseases and Clinical Microbiology Inonu University Faculty of Medicine Malatya Türkiye; ^2^ Department of Infectious Diseases and Clinical Microbiology Dokuz Eylul University Faculty of Medicine Izmir Türkiye; ^3^ Department of Infectious Diseases and Clinical Microbiology Ondokuz Mayis University Faculty of Medicine Samsun Türkiye; ^4^ Department of Infectious Diseases and Clinical Microbiology Ankara Bilkent City Hospital Ankara Türkiye; ^5^ Department of Infectious Diseases and Clinical Microbiology Izmir City Hospital Izmir Türkiye; ^6^ Department of Infectious Diseases and Clinical Microbiology Adana City Training and Research Hospital Adana Türkiye; ^7^ Department of Infectious Diseases and Clinical Microbiology Ankara University Faculty of Medicine Ankara Türkiye; ^8^ Department of Infectious Diseases and Clinical Microbiology Kosuyolu High Specialization Training and Research Hospital Istanbul Türkiye; ^9^ Department of Infectious Diseases and Clinical Microbiology Ankara Etlik City Hospital Ankara Türkiye; ^10^ Department of General Surgery Izmir City Hospital Izmir Türkiye; ^11^ Department of Internal Medicine Dokuz Eylul University Faculty of Medicine Izmir Türkiye; ^12^ Department of General Surgery and Transplantation Institute Inonu University Faculty of Medicine Malatya Türkiye; ^13^ Department of Infectious Diseases and Clinical Microbiology Guven Hospital Ankara Türkiye; ^14^ Department of Infectious Diseases and Clinical Microbiology Baskent University Faculty of Medicine Ankara Türkiye

**Keywords:** advanced age, mortality, multidrug‐resistant organisms, posttransplant infections, solid organ transplantation

## Abstract

**Background:**

In recent years, with the aging population worldwide, the number of elderly undergoing solid organ transplantation has been steadily increasing. However, data on the effects of advanced age on infection characteristics and clinical outcomes in transplant recipients are limited. This study aimed to evaluate the effects of advanced age on infections and clinical outcomes in solid organ transplant recipients.

**Methods:**

This retrospective multicenter cohort study included patients who underwent solid organ transplantation at different transplant centers in Türkiye between 2003 and 2023 and were followed up for at least 1 year. Patients were divided into two age groups: ≥ 60 years and < 60 years. Demographic characteristics, laboratory parameters, infection episodes, and clinical outcomes were compared between the two age groups.

**Results:**

The study included 273 (27.7%) patients aged ≥ 60 years and 712 (72.3%) patients aged < 60 years, totaling 985 recipients. Male sex and liver transplantation were more common in the older age group (*p* < 0.001). Hospital stay was significantly longer in patients aged ≥ 60 years (*p* = 0.002). However, there was no difference between the two age groups in terms of time to first infection or number of infection episodes. The mortality rate in patients aged ≥ 60 years was 21.6%. The most common cause of death was bacterial infection, with carbapenem‐resistant pathogens detected in a substantial proportion of cases.

**Conclusions:**

Although infection incidence was similar across age groups, solid organ transplant recipients aged ≥ 60 years had higher mortality and longer hospital stays, highlighting the need for careful clinical monitoring in older transplant recipients.

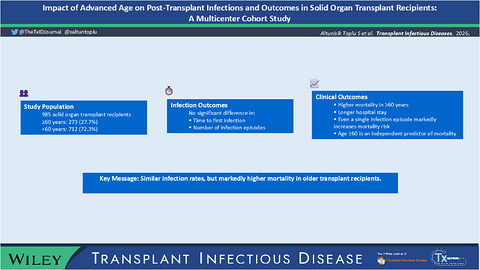

## Graphical abstract

Advanced age (≥60 years) was independently associated with higher mortality in solid organ transplant recipients, despite similar infection incidence. Even a single infection episode significantly increased mortality risk, underscoring the need for close clinical monitoring in elderly patients.

## 1 Introduction

With the aging of the global population, the average age of patients undergoing solid organ transplantation is increasing. Thanks to advances in surgical techniques, progress in immunosuppressive therapy, and improvements in transplant care, solid organ transplantation has become increasingly common in elderly patients [1, 2].

Posttransplant infections are one of the most important causes of morbidity and mortality in solid organ transplant recipients. Infections, especially those developing in the early posttransplant period, have been shown to be associated with graft loss, prolonged hospital stay, and mortality [3].

In recent years, one of the most important problems that complicates infection management in transplant recipients is the increasing frequency of multidrug‐resistant (MDR) microorganisms. The global increase in antimicrobial resistance makes it difficult to treat infections in transplant recipients receiving immunosuppressive therapy and can negatively affect clinical outcomes [4].

Studies on the frequency and clinical outcomes of infections in elderly transplant recipients are limited, and existing studies report conflicting results. While some studies report a higher risk of infection in elderly transplant recipients, other studies show that transplant outcomes can be similar to those of younger recipients when appropriate patient selection is made [5]. Therefore, a better understanding of the epidemiology and clinical outcomes of infections in elderly transplant recipients is important. Türkiye is one of the countries where solid organ transplantation is widely performed and where antimicrobial resistance rates are high. However, multicenter studies evaluating infections and clinical outcomes in elderly transplant recipients in our country are limited. This study aims to present multicenter data evaluating the effect of infections on clinical outcomes in elderly transplant recipients.

## 2 Methods

This study is a retrospective multicenter cohort study evaluating data from different transplant centers in Türkiye. Adult patients who underwent solid organ transplantation between 2003 and 2023 and were followed up for at least 1 year posttransplant were included. Patients were divided into two age groups: ≥ 60 years and < 60 years. To ensure adequate representation of older recipients, approximately two transplant recipients younger than 60 years were included for each patient aged ≥ 60 years.

Demographic characteristics (age, gender), type of transplanted organ, donor type (living or deceased), use of immunosuppressive therapy, and clinical outcomes were retrospectively obtained from patient files and hospital information systems. Pretransplant laboratory parameters included leukocyte, lymphocyte, C‐reactive protein (CRP), creatinine, aspartate aminotransferase (AST), alanine aminotransferase (ALT), gamma‐glutamyl transferase (GGT), lactate dehydrogenase (LDH), and alkaline phosphatase (ALP) values. In addition, the time to the first infection after transplant, the number of infection episodes, and laboratory parameters measured during infection episodes were recorded.

In patients who died, the causative agents of the infection and accompanying clinical conditions were also analyzed. Infection episodes were evaluated based on clinical findings and microbiological data. If a patient developed more than one infection, each infection was recorded as a separate attack. Causes of mortality were classified according to available clinical records and microbiological data.

The distribution of demographic and clinical variables is presented as number (*n*) and percentage (%). The normality of continuous variables was assessed using graphical methods and the Shapiro–Wilk test. Descriptive statistics are presented as mean ± standard deviation. The chi‐square test was used to compare categorical variables across age groups, while the independent samples *t*‐test was used to compare continuous variables. Statistical analyses were performed using IBM SPSS Statistics 26.0 and Microsoft Excel 2024, and the statistical significance level was accepted as *p* < 0.05.

Univariable and multivariable logistic regression analyses were performed to identify independent predictors of mortality, and results were reported as odds ratios (ORs) with 95% confidence intervals (CIs).

The study protocol was approved by the local institutional ethics committee (Approval No: 2025/7462; July 8, 2025). The study was conducted in accordance with the principles of the Declaration of Helsinki. Patient data were retrospectively collected and anonymized before analysis.

## 3 Results

A total of 985 solid organ transplant recipients were included in the study, consisting of 273 patients aged ≥ 60 years (27.7%) and 712 patients aged < 60 years (72.3%).

When demographic and clinical characteristics were compared according to age groups, a statistically significant difference was observed in terms of sex distribution (*p* < 0.001). While 51.3% of patients aged < 60 years were male, 67.4% of patients aged ≥ 60 years were male. There was also a significant difference between the two groups with respect to the type of transplanted organ (*p* < 0.001). Kidney transplantation was more common in the < 60 years group, whereas liver transplantation was more frequent in the ≥ 60 years group.

When donor type was evaluated, the proportion of living donors was significantly higher in patients aged ≥ 60 years (*p* = 0.001). In contrast, steroid use was more common in patients aged < 60 years (*p* < 0.001). In addition, the length of hospital stay was significantly longer in patients aged ≥ 60 years (*p* = 0.002). The detailed distribution of demographic and clinical characteristics is presented in Table 1.

**TABLE 1:** Baseline demographic and clinical characteristics according to age group.

Variable	< 60 years (*n* = 712)	≥ 60 years (*n* = 273)	*p* value
Age (years), mean ± SD	42.39 ± 11.87	66.18 ± 4.28	< 0.001
Sex, *n* (%)			
Male	365 (51.3)	184 (67.4)	< 0.001
Female	347 (48.7)	89 (32.6)	
Transplanted organ, *n* (%)			
Liver	122 (17.3)	100 (36.6)	< 0.001
Kidney	589 (82.9)	168 (61.5)	
Lung	0 (0.0)	5 (1.8)	
Donor type, *n* (%)			
Living donor	345 (51.3)	138 (63.9)	0.001
Deceased donor	328 (48.7)	78 (36.1)	
Steroid use, *n* (%)	687 (97.4)	186 (82.3)	< 0.001
Mortality, *n* (%)	104 (14.6)	59 (21.6)	0.008
Length of hospital stay, mean ± SD	17.98 ± 14.53	21.45 ± 14.22	0.002

When pretransplant laboratory parameters were compared between age groups, statistically significant differences were observed in CRP, creatinine, AST, ALT, GGT, and LDH levels (*p* < 0.05). Mean AST, ALT, GGT, and LDH levels were higher in patients aged ≥ 60 years, whereas CRP and creatinine levels were lower. In contrast, no statistically significant differences were observed in leukocyte, lymphocyte, and ALP levels between the two groups. These findings are presented in Table 2.

**TABLE 2:** Comparison of pretransplant laboratory parameters according to age group.

Parameter	< 60 years (*n* = 712) mean ± SD	≥ 60 years (*n* = 273) mean ± SD	*p* value
Leukocyte	6.84 ± 3.08	7.33 ± 3.97	0.092
Lymphocyte	1.16 ± 0.97	1.27 ± 1.14	0.154
CRP	14.38 ± 17.01	8.92 ± 17.02	0.003
Creatinine	5.63 ± 3.19	2.89 ± 2.67	< 0.001
AST	34.51 ± 70.78	106.37 ± 274.72	< 0.001
ALT	30.99 ± 102.25	79.14 ± 147.02	< 0.001
GGT	46.01 ± 55.44	81.11 ± 121.55	< 0.001
LDH	247.67 ± 120.15	313.16 ± 364.02	0.033
ALP	158.08 ± 195.17	145.91 ± 160.05	0.513

When the time to first infection after transplantation was evaluated, no statistically significant difference was observed between the two age groups (*p* = 0.133). The mean time to first infection was 57.62 ± 76.89 days in patients aged < 60 years and 85.04 ± 195.85 days in patients aged ≥ 60 years. These data are shown in Table 3.

**TABLE 3:** Comparison of time to first infection after transplantation according to age group.

Parameter	< 60 years (*n* = 712) mean ± SD	≥ 60 years (*n* = 273) mean ± SD	*p* value
Time to first infection (days)	57.62 ± 76.89	85.04 ± 195.85	0.133

Laboratory parameters measured during the first infection episode after transplantation were also compared between the age groups. A statistically significant difference was observed in lymphocyte and GGT levels (*p* < 0.05), with higher mean values in patients aged ≥ 60 years. However, no significant differences were found in leukocyte, CRP, creatinine, AST, ALT, LDH, and ALP levels between the two groups. These findings are presented in Table 4.

**TABLE 4:** Comparison of laboratory parameters during the first infection episode after transplantation.

Parameter	< 60 years (*n* = 712) mean ± SD	≥ 60 years (*n* = 273) mean ± SD	*p* value
Leukocyte	8.96 ± 5.83	9.79 ± 5.76	0.141
Lymphocyte	0.89 ± 0.96	1.75 ± 2.69	< 0.001
CRP	59.82 ± 65.28	63.65 ± 87.43	0.648
Creatinine	1.91 ± 1.86	1.69 ± 1.25	0.200
AST	66.11 ± 294.73	98.25 ± 341.19	0.309
ALT	74.54 ± 259.65	115.94 ± 335.32	0.155
GGT	108.65 ± 165.61	147.29 ± 190.62	0.047
LDH	299.33 ± 220.56	292.83 ± 215.61	0.808
ALP	139.92 ± 132.69	165.42 ± 205.08	0.181

Abbreviations: CRP, ALP, alkaline phosphatase; ALT, alanine aminotransferase; AST, aspartate aminotransferase; C‐reactive protein; GGT, gamma‐glutamyl transferase; LDH, lactate dehydrogenase.

When the number of infection episodes was evaluated according to age groups, no statistically significant difference was found between the groups (*p* = 0.707). In the < 60 years group, 62.2% of patients had no infection episodes, 33.3% had one episode, and 4.5% had two or more infection episodes. Similarly, in the ≥ 60 years group, 59.3% of patients had no infection episodes, 35.9% had one episode, and 4.8% had two or more infection episodes. These data are presented in Table S1.

Mortality occurred in 21.6% (*n* = 59) of patients aged ≥ 60 years. The mean postoperative time to mortality was calculated as 73.27 ± 103.27 days. Among patients who died, 59.3% died during the first infection episode. The mortality rate was 62.7% among patients who experienced two or more infection episodes within the first year.

When the causes of mortality were evaluated, bacterial infections were the most common cause (52.5%). Among bacterial pathogens, carbapenem‐resistant bacteria were the most frequently identified (32.2%). Hypertension (27.2%), cardiovascular disease (18.6%), and diabetes (13.6%) were common comorbid conditions among patients who died. These findings are presented in Table S2.

Among patients aged ≥ 60 years who developed mortality, 64.4% were receiving more than one immunosuppressive therapy. In addition, 11.9% were receiving mycophenolate, 5.1% tacrolimus, and 1.7% mTOR inhibitors. No immunosuppressive therapy was used in 16.9% of the patients (Table S3).

Overall, hospital length of stay and mortality rates were higher in transplant recipients aged ≥ 60 years. However, no significant differences were observed between the two age groups in terms of time to infection development and the number of infection episodes.

To further evaluate factors associated with mortality, univariable and multivariable logistic regression analyses were performed.

In univariable analysis, age group, sex, transplant type, steroid use, number of infection episodes, AST, ALT, and GGT levels were significantly associated with mortality (*p* < 0.05). However, leukocyte, lymphocyte, CRP, creatinine levels, and time to first infection were not significantly associated with mortality. In multivariable logistic regression analysis, age ≥ 60 years, steroid use, and infection episodes remained independently associated with mortality. Patients aged ≥ 60 years had a significantly higher risk of mortality (OR: 2.48; 95% CI: 1.27–4.83; *p* = 0.008). Steroid use was associated with a reduced risk of mortality (OR: 0.31; 95% CI: 0.13–0.71; *p* = 0.006). In addition, patients who experienced at least one infection episode had a markedly increased risk of mortality (OR: 4.39; 95% CI: 2.35–8.20; *p* < 0.001). Variables such as sex, transplant type, and liver enzyme levels lost their significance in the multivariable model, suggesting that their effects may be confounded by other clinical factors (Table S4).

Additional detailed analyses are provided in the Supporting Information.

## 4 Discussion

This multicenter study evaluated the effect of advanced age on infections and clinical outcomes in solid organ transplant recipients. One of the key findings of our study was that although transplant recipients aged 60 and over had longer hospital stays and higher mortality rates, there was no significant difference between the two age groups in terms of the time to infection development and the number of infection episodes. These findings suggest that the frequency of infections in older transplant recipients may be similar to that in younger recipients, but the clinical outcomes of infections may be more severe.

One of the most important findings of our study is that advanced age remained an independent risk factor for mortality even after adjustment for potential confounders. This suggests that the increased mortality observed in elderly transplant recipients cannot be explained solely by differences in transplant type or baseline characteristics.

In addition, the strong association between infection episodes and mortality highlights the critical role of infection burden in determining outcomes. Notably, the finding that even a single infection episode significantly increases mortality risk underscores the importance of early detection and aggressive management of infections in this population.

Interestingly, steroid use was associated with reduced mortality in the multivariable analysis. This finding may reflect the role of optimized immunosuppressive regimens in maintaining graft function and preventing severe inflammatory complications, although residual confounding cannot be excluded.

In recent years, it has been reported that the average age of patients undergoing solid organ transplantation is increasing worldwide due to the aging population [6, 7]. Thanks to advances in transplant surgery and the effectiveness of immunosuppressive therapies, transplantation has become more frequently performed in elderly patients [8]. However, the clinical outcomes of elderly transplant recipients remain a controversial topic in the literature. While some studies report higher infection and mortality rates in elderly transplant recipients, other studies have shown that transplant outcomes can be similar to those of younger recipients when appropriate patient selection is made [9, 10, 11].

Some studies in the literature have reported that infections may have a more significant impact on clinical outcomes in elderly transplant recipients [12]. In particular, large cohort studies in kidney transplant recipients have shown that infections are strongly associated with graft loss and mortality [13, 14]. Similarly, in our study, higher mortality rates were observed in the older age group, and this finding is consistent with the results reported in the literature.

The finding that the frequency of infections in elderly transplant recipients is similar to that in younger recipients is consistent with some studies reported in the literature [15, 16]. Although it is thought that the risk of developing infections may increase in older patients, the careful selection of transplant candidates and close clinical monitoring after transplantation may contribute to the infection frequency being at levels similar to that in younger patients. However, the clinical course of infections can be more severe in elderly transplant recipients, and clinical outcomes may be more unfavorable when infection develops. Although no significant difference was found between the two age groups in terms of the time of infection development and the number of infection episodes in our study, the higher mortality rates in the older age group support this situation.

One of the important factors determining the impact of infections on clinical outcomes in transplant recipients is the presence of MDR microorganisms. In recent years, infections caused by MDR bacteria have been strongly associated with mortality in solid organ transplant recipients [4]. In particular, carbapenem‐resistant Gram‐negative bacteria, methicillin‐resistant *Staphylococcus aureus* (MRSA), and vancomycin‐resistant enterococci (VRE) are among the main pathogens that can cause difficult‐to‐treat infections in transplant recipients [17, 18, 19]. In our study, bacterial infections were prominent in patients who developed mortality, and a significant portion of these infections were associated with carbapenem‐resistant bacteria. This situation once again highlights the impact of antimicrobial resistance on clinical outcomes in transplant recipients.

This multicenter study provides valuable data on infections and clinical outcomes associated with advanced age in solid‐organ transplant recipients. The inclusion of data from multiple transplant centers and a relatively large patient population increases the generalizability of the results. However, due to the retrospective design of the study, some potentially relevant clinical variables could not be fully evaluated. In addition, the long study period may have included temporal changes in antimicrobial resistance patterns, which were not specifically analyzed in this study. Furthermore, the lack of detailed subgroup analyses according to transplant type and the absence of some clinical variables in the available dataset represent additional limitations of the study.

In conclusion, although the incidence of infections was similar across age groups, advanced age remained an independent predictor of mortality in solid organ transplant recipients. Notably, even a single infection episode was associated with a significantly increased risk of mortality, underscoring the critical importance of early recognition and prompt management of infections in elderly patients.

## Author Contributions

**Vildan Avkan Oguz**: data curation, supervision, writing – review and editing, resources, writing – original draft, methodology, conceptualization, visualization, investigation, validation. **Sibel Altunisik Toplu**: conceptualization, methodology, writing – original draft, writing – review and editing, visualization, formal analysis, resources, data curation, investigation, validation, project administration. **Esra Tanyel**: data curation, investigation, writing – original draft, writing – review and editing. **Bengu Tatar**: data curation, investigation. **Adam Uslu**: data curation, investigation. **Imran Hasanoglu**: data curation, investigation, methodology, writing – original draft, writing – review and editing. **Nilay Danis**: data curation, investigation. **Adem Kose**: data curation, investigation. **Gulnur Kul**: data curation, investigation. **Tuba Turunc**: data curation, investigation, writing – original draft, writing – review and editing. **Hande Arslan**: data curation, methodology. **Yesim Uygun Kizmaz**: data curation, investigation. **Ezgi Erdal Karakas**: data curation, investigation. **Yasar Bayindir**: data curation, methodology. **Ebru Oruc**: data curation. **Muammer Celik**: data curation, investigation. **Sezai Yilmaz**: data curation. **Yelda Deligoz Bildaci**: data curation, investigation. **Gule Cinar**: data curation, investigation, writing – original draft.

## Consent

The authors have nothing to report.

## Supporting information




**Supplementary Table 1**: Comparison of Number of Infection Episodes According to Age Group.
**Supplementary Table 2**: Mortality Characteristics in Patients Aged ≥60 Years.
**Supplementary Table 3**: Immunosuppressive Therapy in Patients Aged ≥60 Years With Mortality.
**Supplementary Table 4**: Univariable and Multivariable Logistic Regression Analysis of Factors Associated with Mortality.

**Supplementary**: tid70232‐sup‐0002‐VisualAbstract.tif.
