# Supplementary material for: Impact of Advanced Age on Posttransplant Infections and Outcomes in Solid Organ Transplant Recipients: A Multicenter Cohort Study
Source: Transpl Infect Dis. 2026 May 6;28(4):e70232. doi: 10.1111/tid.70232 (PMC13450045; doi:10.1111/tid.70232)
Supplement: Supplementary file 1 [file TID-28-e70232-s001.docx]

**Supplementary Table 1. Comparison of Number of Infection Episodes According to Age Group**

| **Number of infection episodes** | **<60 years (n=712) n (%)** | **≥60 years (n=273) n (%)** | **p value** |
| --- | --- | --- | --- |
| None | 443 (62.2) | 162 (59.3) | 0.707 |
| One episode | 237 (33.3) | 98 (35.9) |  |
| Two or more episodes | 32 (4.5) | 13 (4.8) |  |

**Supplementary Table 2. Mortality Characteristics in Patients Aged ≥60 Years**

| **Variable** | **≥60 years (n=273) n (%)** |
| --- | --- |
| Mortality | 59 (21.6) |
| Time to mortality (days), mean ± SD | 73.27 ± 103.27 |
| Mortality during first infection episode | 35 (59.3) |
| Mortality in patients with ≥2 infection episodes within first year | 37 (62.7) |
| Pathogens |  |
| Bacterial | 31 (52.5) |
| Viral | 0 (0.0) |
| Fungal | 2 (3.4) |
| No pathogen identified | 26 (44.1) |
| Bacterial species |  |
| Carbapenem-resistant bacteria | 10 (32.2) |
| MRSA | 2 (6.4) |
| VRE | 1 (3.2) |
| Other bacteria | 18 (58.1) |
| Candida species | 2 (100.0) |
| Coinfection | 1 (1.7) |
| Diabetes mellitus | 8 (13.6) |
| Hypertension | 16 (27.2) |
| Cardiovascular disease | 11 (18.6) |
| Rejection | 6 (10.2) |
| Graft loss | 5 (8.5) |

**Abbreviations:**
MRSA, methicillin-resistant *Staphylococcus aureus*; VRE, vancomycin-resistant *Enterococcus*.

**Supplementary Table 3. Immunosuppressive Therapy in Patients Aged ≥60 Years With Mortality**

| **Immunosuppressive therapy** | **n (%)** |
| --- | --- |
| Mycophenolate | 7 (11.9) |
| Tacrolimus | 3 (5.1) |
| mTOR inhibitor | 1 (1.7) |
| Multiple immunosuppressive agents | 38 (64.4) |
| No immunosuppressive therapy | 10 (16.9) |

**Supplementary Table 4.** Univariable and Multivariable Logistic Regression Analysis of Factors Associated with Mortality

|  | **Univariable** | | **Multivariable** | |
| --- | --- | --- | --- | --- |
|  | **p** | **OR (95% CI)** | **p** | **OR (95% CI)** |
| Age group (≥60 years) | **0,008** | 1.612 (1.130–2.300) | **0.008** | 2.480 (1.273–4.831) |
| Sex (female) | **0.027** | 1,462 (1.044–2.048) | 0.188 | 1.522 (0.815–2.843) |
| Transplant type (kidney) | **0,041** | 0.674 (0.462–0.983) | 0.282 | 0.602 (0.239–1.518) |
| Donor type (deceased donor) | 0.195 | 1.255 (0.890–1.769) | 0.068 | 1.841 (0.955–3.548) |
| Steroid use | **<0.001** | 0.288 (0.165–0.503) | **0.006** | 0.307 (0.132–0.713) |
| Length of hospital stay | 0.083 | 1.009 (0.99–1.020) | 0.990 | 1.000 (0.979–1.021) |
| Infection episodes | **<0.001** |  | **<0.001** |  |
| One episode | **<0.001** | 3.233 (2.280–4.583) | **<0.001** | 4.394 (2.354–8.203) |
| ≥2 episodes | **0.188** | 0.380 (0.090–1.604) | 0.998 | 0.001 (0.001–0.001) |
| Leukocyte | 0.382 | 1.022 (0.973–1.074) |  |  |
| Lymphocyte | 0.408 | 0.924 (0.766–1.114) |  |  |
| CRP | 0.639 | 1.004 (0.987–1.022) |  |  |
| Creatinine | 0.051 | 0.948 (0.898–1.000) | 0.505 | 1.052 (0.907–1.221) |
| AST | **0.004** | 1.002 (1.001–1.004) | 0.147 | 1.003 (0.999–1.007) |
| ALT | **0.021** | 1.002 (1.001–1.003) | 0.580 | 0.999 (0.995–1.003) |
| GGT | **0.031** | 1.003 (1.001–1.005) | 0.331 | 1.001 (0.999–1.004) |
| Time to first infection | 0.284 | 0.999 (0.996–1.001) |  |  |

OR: Odds ratio. CI, confidence interval. CRP, C-reactive protein; AST, aspartate aminotransferase; ALT, alanine aminotransferase; GGT, gamma-glutamyl transferase. Variables included in the multivariable logistic regression model were selected from those with a p-value <0.20 in univariable analysis.
